# Reversible Myocardial Injury and Intraventricular Thrombus Associated with Aluminium Phosphide Poisoning

**DOI:** 10.1155/2017/6287015

**Published:** 2017-04-09

**Authors:** Abdelkader Jalil El Hangouche, Hala Fennich, Oumaima Alaika, Taoufiq Dakka, Zaineb Raissouni, Latifa Oukerraj, Nawal Doghmi, Mohamed Cherti

**Affiliations:** ^1^Laboratory of Physiology, Faculty of Medicine and Pharmacy of Tangier, Abdelmalek Essaâdi University, Tangier, Morocco; ^2^Department of Cardiology B, Ibn Sina Hospital, Mohammed V University, Rabat, Morocco; ^3^Laboratory of Physiology, Faculty of Medicine and Pharmacy of Rabat, Mohammed V University, Rabat, Morocco

## Abstract

Aluminium phosphide (ALP) is widely used as a fumigant pesticide. In case of ALP poisoning, it is responsible for myocardial dysfunction, related to toxic myocarditis, and hemodynamic disorders. We report a case of a 28-year-old female who had intentionally ingested ALP and was admitted with cardiogenic shock. The transthoracic echocardiography (TTE) at the time of admission showed severe global myocardial hypokinesia with the presence of a giant left ventricular thrombus. Cardiovascular magnetic resonance (CMR) revealed extensive toxic myocarditis with a left ventricular systolic dysfunction. All cardiac lesions were reversible after symptomatic treatment, within 6 months. We aim, by reporting this case, to evidence the complete reversibility of cardiac injury due to aluminium phosphide poisoning documented by transthoracic echocardiography and cardiovascular magnetic resonance.

## 1. Introduction

In Morocco, ALP poisoning is a serious healthcare problem [1], with a high mortality despite the progress of critical care. Most poisoned patients die from refractory cardiac shock [2].

Few cases of reversible myocardial injury have been described in the literature. None of them has been evaluated by cardiovascular magnetic resonance [2].

## 2. Case Presentation

A 28-year-old woman was admitted to the emergency department with profuse sweating and dizziness, two hours following intentional ingestion of 1 tablet of aluminium phosphide, of which the confirmation was based on toxic ingestion history reported by the case's relatives. She was immediately transferred to the intensive care unit and received gastric lavage.

Physical examination at the time of admission revealed general malaise, pulse rate of 120 bpm, blood pressure of 100/60 mmHg, and oxygen saturation of 97% on room air (pulse oximetry).

**Figure 1:**
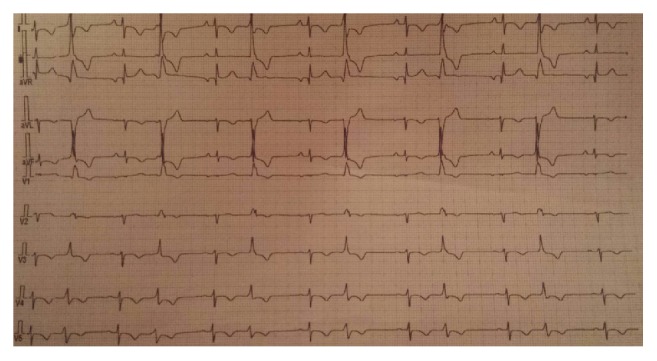
Electrocardiogram shows normal sinus rhythm, ST elevation in AVr, ST depression, and T waves inversion in inferior and apicolateral leads with premature ventricular beats.

Chest X-ray was normal. The electrocardiogram showed ST-segment elevation in AVr lead with ST-segment depression in inferior and anteroapical leads (Figure 1).

Blood analysis revealed increased cardiac troponin I concentration of 17.5 ng/mL (50 times the ULN), glycemia of 0.90 g/dL, sodium of 140 mEq/L, and potassium of 3.8 mEq/L.

One hour after admission, the patient developed signs of cardiogenic shock: hypotension (85/40 mmHg), sinus tachycardia (142 bpm), polypnea (28 cycles/min), and GCS score of 12.

The patient was assisted by mechanical ventilation and intravenous inotropic and vasopressor agents (dobutamine 20 *μ*g/kg/min, dopamine 20 *μ*g/kg/min) with remarkable hemodynamic improvement 24 hours later.

**Figure 2:**
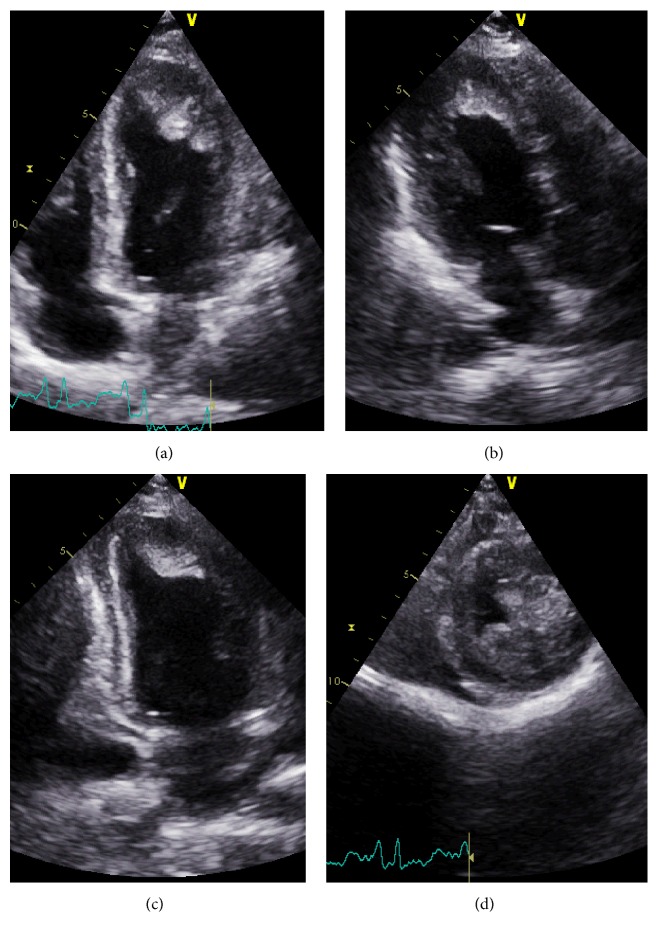
Transthoracic echocardiography showing apical left ventricular thrombus extending to the anterior and anterolateral walls. (a) Apical four-chamber view; (b) apical three-chamber view; (c) apical two-chamber view; (d) short axis view at the apical level.

**Figure 3:**
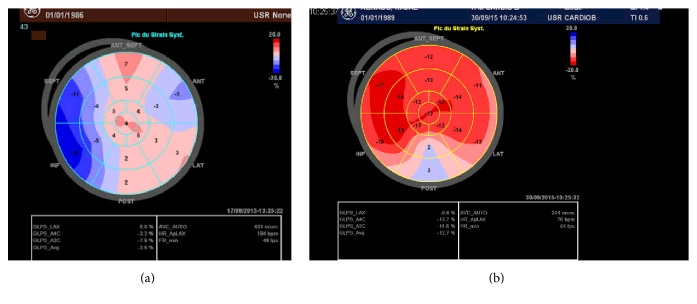
(a) Global longitudinal strain of the left ventricule at −3.5% suggesting severely reduced left ventricle systolic function. (b) Global longitudinal strain improved to −13.5% after 25 days.

**Figure 4:**
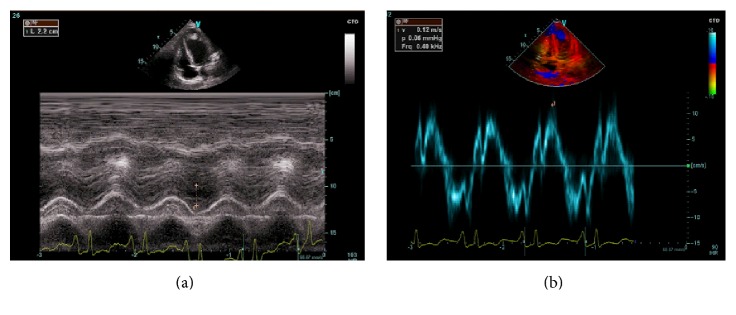
Transthoracic echocardiography, tissular Doppler showing a preserved function of the right ventricule: (a) TAPSE at 22 mm, (b) tricuspid lateral annular systolic velocity wave (S′) at 0.12 m/s.

On the second day of intoxication, a transthoracic echocardiography was performed and objectified global hypokinesia of the left ventricule with an ejection fraction of 20% and an apical left ventricular thrombus extending to the anterior and anterolateral walls measuring 25 × 30 mm (Figure 2). The global longitudinal strain of the left ventricule was evaluated at −3.5% (Figure 3(a)). The right ventricule had a preserved systolic function (Figure 4).

Regarding the echocardiographic findings and after clinical stabilization, an adjuvant treatment including diuretics (furosemide 40 mg per day), beta-blockers (carvedilol 12 increased to 25 mg twice daily), and spironolactone (25 mg increased to 50 mg per day) was introduced.

**Figure 5:**
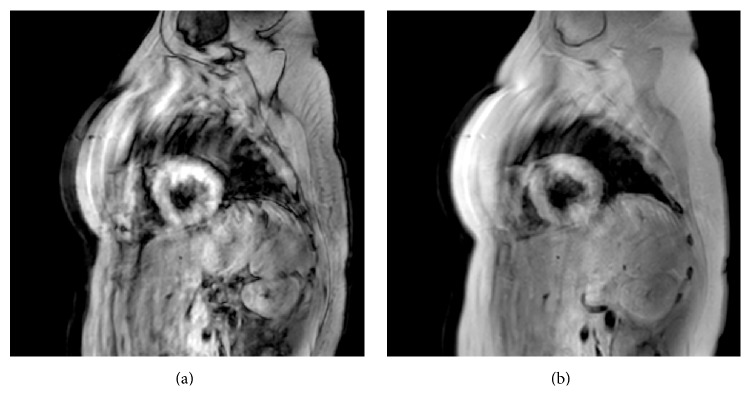
Cardiac magnetic resonance imaging: sequences HASTE (short axis) showing a hypersignal in the anterior wall (a) on the 5th day and (b) on the 60th day of aluminium phosphide poisoning.

**Figure 6:**
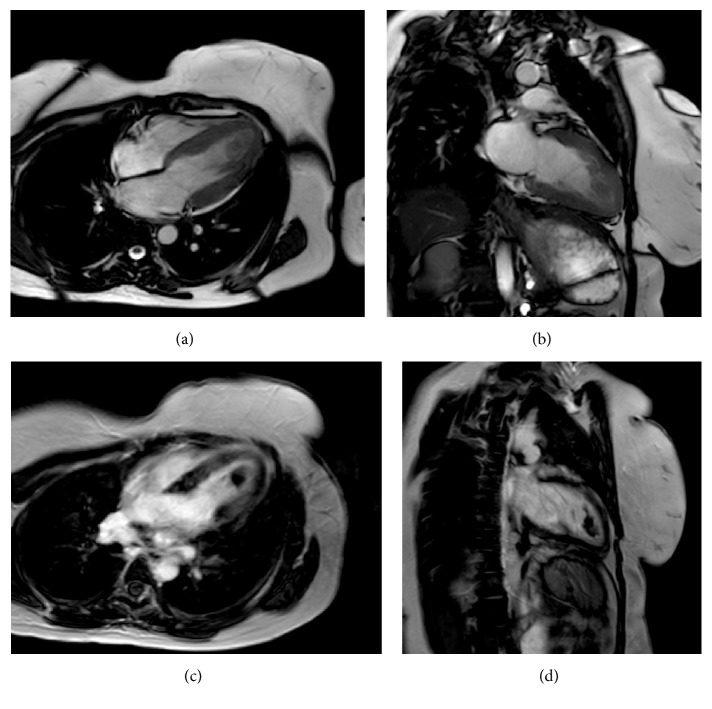
Cardiac magnetic resonance imaging showing a thrombus in the left ventricule apex and late gadolinium enhancement (LGE). (a) Four-chamber view: ventricular thrombus measuring 25 × 30 mm; (b) two-chamber view: ventricular thrombus extending to the anterior wall; (c) four-chamber view (10 min of gadolinium injection): LGE in inferoseptal and anterolateral walls and in the apex; (d) two-chamber view (10 min of gadolinium injection): LGE in the anterior wall and in the apex.

Cardiovascular magnetic resonance was performed using a high performance 1.5-Tesla magnet. It showed an intramyocardial subepicardial signal anomaly in the anterior, inferoseptal, and anterolateral walls in T2 and in late gadolinium enhancement in T1-weighted images (after 10 min of gadolinium injection) suggesting acute toxic myocarditis (Figures 5(a) and 6). Additionally, a left intraventricular thrombus was found.

**Figure 7:**
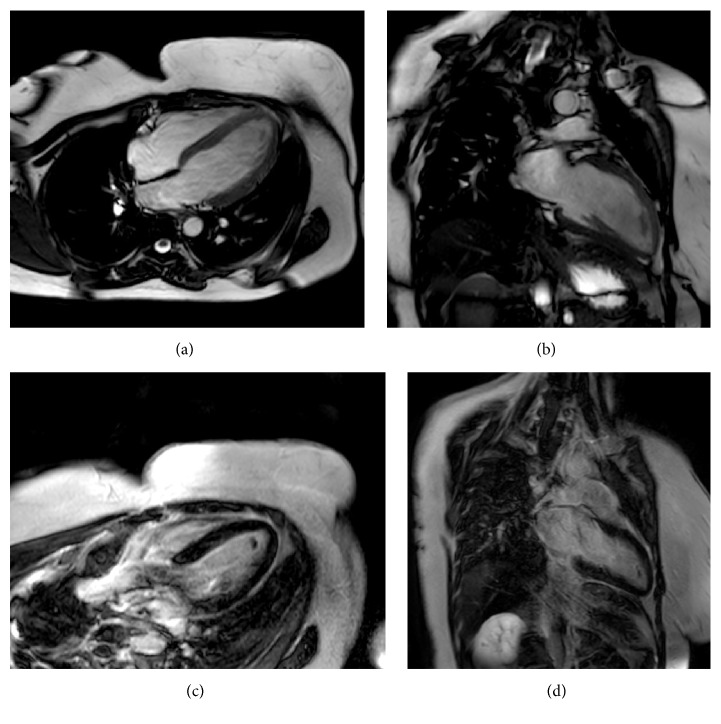
Cardiac magnetic resonance imaging showing a decreased thrombus size in two months following ALP poisoning (after anticoagulant therapy). (a) Four-chamber view and (b) two-chamber view show a decrease of thrombus size of 80%; (c) four-chamber view (10 min of late gadolinium enhancement): regression of LGE in inferoseptal and anterolateral walls and in the apex; (d) two-chamber view (10 min of late gadolinium enhancement): regression of LGE in the anterior wall.

On follow-up, there was progressive improvement of the hemodynamic, biologic, electric, and echocardiographic parameters. Both the left ventricule ejection fraction and the global longitudinal strain increased, respectively, to 55% and to −13.5% (Figure 3(b)), after 25 days of ALP ingestion. Regression of the intraventricular thrombus, of the hyperintense signal in the anterior wall in T2 (Figure 5(b)), and of late gadolinium enhancement in inferoseptal, anterior, and anterolateral walls in T1-weighted images (Figure 7) was confirmed by CMR control in two months.

The patient remains asymptomatic at one-year follow-up.

## 3. Discussion

ALP is one of the most abused suicidal chemical agents in Morocco. It is marketed as dark gray tablets of 3 g, consisting of aluminium phosphide (56%) and ammonium carbamate (44%) [3].

When it comes in contact with gastric hydrochloric acid or with atmospheric moisture, ALP releases a lethal phosphine gas [4].

It is a mitochondrial toxin that causes depression of cardiac contractility, secondary to toxic myocarditis, and hemodynamic disorders.

The accurate mechanism of cardiotoxicity of ALP is not well defined [5]. It includes corrosive action of phosphine, inhibition of cytochrome C oxidase, and formation of highly reactive hydroxyl radicals [6]. Indeed, indicators of oxidative stress reach peak levels within 48 h of exposure to the poison, approaching normalization on the fifth day [7].

The clinical presentation depends on the amount of toxin ingested, the route of administration, and the time elapsed since exposure to the poison [8].

Within a few minutes following the toxic ingestion, the patient develops symptoms of cardiovascular, gastrointestinal, respiratory, and nervous systems deterioration. Then, a few hours later, symptoms of hepatic and renal failure and disseminated intravascular coagulation appear [9].

The acute cardiovascular collapse secondary to phosphine-induced myocardial damage is the most common mode of presentation (60–100% of cases) [8].

Several studies noted electric abnormalities, as conduction disorders, cardiac arrhythmias, and nonspecific ST-T wave changes, resulting from focal myocardial necrosis and changes in membrane action potentials [9]. Singh et al. reported that electrocardiogram changes revert to normal pattern within 10–14 days in survivors [10].

The transthoracic echocardiogram of our case showed an important reduction in the left ventricular systolic function with global severe hypokinesia. Similar cases have been reported with a significant decrease in the left ventricular ejection fraction. Bhasin et al. demonstrated a similar pattern of global hypokinesia of the left ventricule walls in 80% of their cases. Elabbassi et al. had reported normalization of echocardiographic findings in aluminium phosphide poisoning survivors by the fifth day [11]. Concerning our case, the TTE control showed a gradual improvement in the left ventricular ejection fraction by the 14th day.

To our knowledge, we are reporting the first case of left intraventricular thrombus of ALP poisoning.

Cardiovascular magnetic resonance, a noninvasive, nonradiating technique, has been successfully used in the evaluation of both ischemic and nonischemic heart diseases. The ability to detect early myocardial changes missed by other imaging techniques, using tissue characterization, makes CMR an excellent diagnostic tool for early and detailed assessment of cardiovascular diseases. Additionally, CMR is the best noninvasive tool currently used for the detection of myocarditis. It has become actually the leading noninvasive imaging modality of myocarditis. The consensual use of the 3 criteria “Lake Louise Criteria” (signal intensity increase in T2-weighted images “edema,” early gadolinium enhancement in T1-weighted images “hyperemia,” and late gadolinium enhancement in T1-weighted images “necrosis and fibrosis”) has standardized the CMR protocol for myocarditis diagnosis [12].

In our case, CMR showed myocardial edema in the T2-weighted images and myocardial areas of late gadolinium enhancement in the early phase. Follow-up CMR in two months showed favourable evolution with regression of the above finding. Furthermore, CMR provided valuable information about the systolic function of both right and left ventricles and the left intraventricular thrombus. To our knowledge, this is the first report describing finding of CMR in ALP ingestion.

Since there is no specific antidote to ALP poisoning, the management of ALP intoxication remains primarily supportive care. The use of different therapeutic modalities was reported in the literature with varied degrees of success, including magnesium sulphate [13], N-acetyl cysteine [14], triiodothyronine [15], intra-aortic balloon pump [16], and extracorporeal membrane oxygenation as an assist device [8].

The majority of poisoned patients die despite intensive medical care [8]. However, our patient survived with no sequelae and remind free of symptoms at one-year follow-up because of symptomatic treatment.

## 4. Conclusion

Myocardial injury following ALP poisoning is responsible for high mortality, mainly due to hemodynamic failure attributable to toxic myocarditis [3]. However, this injury could be reversible few days after intensive medical care as shown in our case. That underlines the interest in the use of transthoracic echocardiography and cardiovascular magnetic resonance to track the progress of myocardial dysfunction and look for thrombotic complications in the early phase of ALP intoxication.
